# Recombinant *Lactococcus lactis* Expressing Ling Zhi 8 Protein Ameliorates Nonalcoholic Fatty Liver and Early Atherogenesis in Cholesterol-Fed Rabbits

**DOI:** 10.1155/2020/3495682

**Published:** 2020-01-26

**Authors:** Mey-Fann Lee, Chu-Hui Chiang, Shyh-Jye Lin, Pei-Pong Song, Hsin-Chun Liu, Tsu-Juey Wu, Wei-Wen Lin

**Affiliations:** ^1^Department of Medical Research, Taichung Veterans General Hospital, Taichung, Taiwan; ^2^Department of Plant Medicine, National Pingtung University of Science and Technology, Pingtung, Taiwan; ^3^School of Medical Laboratory and Biotechnology, Chung Shan Medical University, Taichung, Taiwan; ^4^Cardiovascular Center, Taichung Veterans General Hospital, Taichung, Taiwan; ^5^Institute of Clinical Medicine, and Cardiovascular Research Institute, Department of Medicine, School of Medicine, National Yang-Ming University, Taipei, Taiwan; ^6^Division of Cardiology, Department of Internal Medicine, Taichung Veterans General Hospital, Puli Branch, Taichung, Taiwan; ^7^Department of Life Science, Tunghai University, Taichung, Taiwan

## Abstract

Atherosclerosis is an inflammatory disease characterized by lipid deposits in the subendothelial space leading to severe inflammation. Nonalcoholic fatty liver disease (NAFLD) shares several risk factors with atherosclerosis, including dyslipidemia, type 2 diabetes mellitus, and metabolic syndrome, all of which lead to lipid deposition in the liver causing inflammation and fibrosis. Several clinical trials have shown that certain Chinese herbal medicines with anti-inflammatory effects can be used as adjuvant therapy to prevent the development of cardiovascular events and liver disease. Ling Zhi 8 (LZ8) is an immunomodulatory protein isolated from a medicinal mushroom and has been well documented to possess a broad range of pharmacological properties. This study aimed to evaluate the protective effects of recombinant *Lactococcus lactis* expressing LZ8 protein on NAFLD and atherogenesis in a cholesterol-fed rabbit model. Twelve rabbits were divided into three groups and fed with syrup only, *L. lactis* vehicle, or recombinant *L. lactis*-LZ8 once a day on weekdays for five weeks, respectively. The gene expression of IL-1*β* in the aorta was significantly suppressed after oral administration of *L. lactis*-LZ8. Moreover, in hematoxylin and eosin staining of the aorta, the intima-medial thickness was decreased, and foam cells were significantly reduced in the subendothelial space. LZ8 also inhibited the expression of IL-1*β* in the liver, decreased fat droplet deposits and infiltration of inflammatory cells, and improved liver function by decreasing liver enzymes in an animal model. Our results suggest that the Lactococcus-expressing LZ8 appears to be a promising medicine for improving both NAFLD and early atherogenesis owing to its anti-inflammatory effect. Furthermore, it is available as a low-cost food-grade product.

## 1. Introduction

Atherosclerosis is the major cause of myocardial infarction, stroke, and peripheral artery disease. It is a chronic inflammatory process characterized by low-density lipoprotein cholesterol (LDL-C) accumulation in the subendothelial space [1]. Lipid-lowering therapy with statins is the standard treatment for atherosclerotic patients [2]; however, such patients remain at high risk of recurrent atherosclerosis despite aggressive lipid modification treatment. Nonalcoholic fatty liver disease (NAFLD) shares several risk factors with atherosclerosis, including dyslipidemia, type 2 diabetes mellitus, and metabolic syndrome [3, 4]. NAFLD involves a histopathological spectrum including fat accumulation in hepatocytes with different degrees of inflammation and fibrosis. In a large epidemiology cohort study, over 5000 asymptomatic individuals with no history of coronary artery disease or significant alcohol intake received abdominal ultrasonography and coronary computed tomography angiography in a general health examination [5]. The authors reported that NAFLD was consistently associated with coronary artery soft plaque, suggesting early atherosclerosis [5]. There are many etiological and pathological similarities between atherosclerosis and NAFLD, including hypercholesteremia and hypertriglyceridemia, which lead to lipid deposition in tissue and cause inflammation and fibrosis [6, 7].

Several clinical trials have shown that certain Chinese herbal medicines have anti-inflammatory effects and that these medicines could potentially be used as adjuvant therapy to prevent the recurrence of cardiovascular events and liver disease. Ling Zhi 8 (LZ8) is an immunomodulatory protein isolated from the medicinal mushroom known as Ling Zhi, and its nucleotide sequences and structure have been characterized in several studies [8, 9]. It has been well documented that LZ8 possesses a broad range of pharmacological properties, including anti-inflammatory activities [10–13]. However, few studies have investigated its anti-inflammatory effects on atherosclerosis and NAFLD. Over the past two decades, *Lactococcus lactis* has been used as a food-grade and endotoxin-free genetically engineered vector for protein expression and as an antigen delivery system [14–16]. This study aimed to evaluate the protective effects of recombinant *L. lactis* expressing LZ8 protein on NAFLD and atherogenesis in a cholesterol-fed rabbit model.

## 2. Materials and Methods

### 2.1. Bacterial Strain and Vector

The *Lactococcus lactis* NZ3900 strain and plasmid pNZ8149 were purchased from MoBiTec (Goettingen, Germany). NZ3900 is the standard strain for food-grade selection due to its ability to grow on lactose. pNZ8149 contains the *lacF* gene for food-grade selection for growth on lactose and a nisA promoter for nisin-induced gene expression.

### 2.2. Construction of pNZ8149-LZ8 and Transformation by Electroporation

To construct the recombinant plasmid expressing the fusion gene under the control of the regulatory promoter nisA, the encoding gene of LZ8 from *Ganoderma lucidum* (described in GenBank *M58032.1*) was synthesized by modifying its sequence based on the optimized codon usage in *L. lactis*. 335-bp synthetic fragments of LZ8 were then cloned into the *Nco*I/*Xba*I sites of the pNZ8149 vector in-frame and confirmed by DNA sequencing using an automated DNA analyzer (ABI Prism 3700) (Sequence data shown in Supplement [Supplementary-material supplementary-material-1]). Computer-assisted homology search revealed that these novel LZ sequences had high identities, 70.57% and 100%, with the published LZ8 in nucleotide and amino acid sequences, respectively. The constructed plasmid was transformed into the *L. lactis* strain NZ3900 using a Gene Pulser (2500 V, 200 Ω, 25 *μ*F, 5 ms, Bio-Rad, USA). The electroporated mixture was plated onto Elliker plates according to the manufacturer's instructions (MoBiTec). Lactose-positive colonies appeared yellow after 48 h of incubation at 30°C.

### 2.3. Protein Expression with the Food-Grade Inducer Nisin

The genetically engineered strain NZ3900/pNZ8149-LZ8 was propagated in M17 medium containing 0.5% lactose as the sole carbon source at 30°C. The recombinant clone was grown until an OD_600_ of 0.5 was reached, which was induced with different concentrations of nisin (0∼50 ng/mL, Sigma, Missouri, USA) for 1∼20 h. The corresponding control strain NZ3900 harboring an empty plasmid was treated in the same way. The harvested cells were monitored for protein expression using immunodetection with a lab-made rabbit anti-LZ8 polyclonal antibody as described previously [17]. For oral vaccination, the harvested cell pellets were washed twice with PBS and the expressed protein levels were determined by immunoblotting using purified *E. coli*-derived LZ8 as the quantification standard.

### 2.4. Experimental Design of Oral Live Recombinant LZ8 *L. lactis* and Hypercholesterolemic Rabbit Model

**Figure 1:**
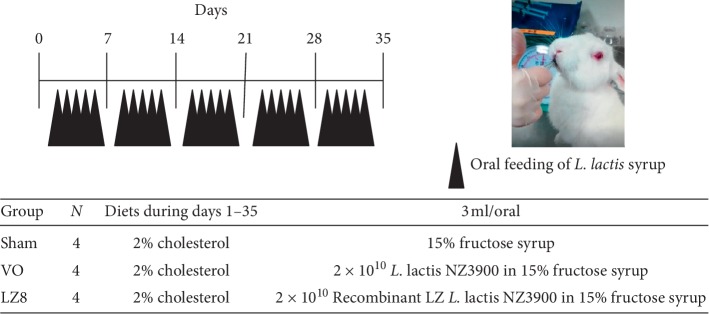
Experimental scheme of oral recombinant *Lactococcus lactis* in rabbits fed with a high cholesterol diet. Twelve male New Zealand white rabbits were divided into three groups and fed 3 ml of the prepared fructose syrup as indicated once a day on weekdays. Blood samples were collected weekly via the marginal ear vein and all rabbits were sacrificed on day 35.

The experimental protocols of oral administration of recombinant *L. lactis* in rabbits fed with a high cholesterol diet are described in Figure 1. Twelve male 2-month-old New Zealand white rabbits (body weight 2.45 ± 0.30 kg) were purchased from a private farm in Changhua, Taiwan, and housed separately in cages. The experimental rabbits received commercial rabbit chow supplemented with 2% cholesterol, 1% cholic acid, and 0.5% thiouracil for 35 days and were then fasted for 4 hours prior to oral *L. lactis*. Briefly, the rabbits were divided into three groups and fed with 3 mL of the prepared 15% fructose syrup as depicted in Figure 1 once a day on weekdays for 5 weeks. Blood samples from the fasted rabbits were collected in no-additive tubes via the marginal ear vein weekly and then centrifuged to separate the serum, which was kept frozen at −20°C until use. After 5 weeks, the rabbits were anesthetized with a mixture of ketamine (40 mg/kg) and xylazine (5 mg/kg) given intramuscularly and then sacrificed. The aortas from the ascending aorta, aortic arch to thoracic aorta, and livers were quickly removed for further analyses.

### 2.5. Lipid Profiles in Sera

To evaluate the effects of oral recombinant LZ8 *L. lactis* on the rabbits, the concentrations of triglycerides (TGs), total cholesterol (LDL-C), high density lipoprotein cholesterol (HDL-C), aspartate transaminase (AST), and alanine transaminase (ALT) were determined in the sera of fasted rabbits using an automated analyzer with commercially available kits.

### 2.6. Sudan Red Staining of Rabbit Aortas

To more accurately analyze intima lipid infiltration, Sudan red staining of aortic intima was performed in six rabbits from the three groups. On day 35, the rabbits were sacrificed and the aortas were dissected and carefully cleaned to remove surrounding tissue. The aortas were then washed with PBS and immersed in Sudan IV stain solution (5% (w/v) Sudan IV in 35% ethanol and 50% acetone) at room temperature for 5 min. The tissues were then placed in 80% ethanol for 3 min and washed in running water. The aortas were then longitudinally cut to expose the inner lumen, pinned on a rubber pad with the interior facing up, and photographed using a digital camera (Sony, Japan).

### 2.7. Hematoxylin and Eosin Staining of Rabbit Livers and Aortas

Twelve liver tissue samples and six aortic arches from the three groups of rabbits were fixed in formalin and embedded in paraffin, dissected into 3 *μ*m sections, deparaffinized with xylene, and then rehydrated in a graded series of ethanol for hematoxylin and eosin (H&E) staining. The extent of atherosclerotic lesions was analyzed by determining the intima to medial thickness ratio histologically using light microscopy, and corresponding images were taken using an Olympus BX51 microscopic/DP71 Digital Camera System (Nagano, Japan).

### 2.8. RNA Extraction and Reverse Transcription-Polymerase Chain Reaction

Total RNA was extracted from rabbit ascending aortas and livers with TRIzol reagent (Invitrogen, CA, USA), and cDNAs were generated from 2 *μ*g of total RNA using a SuperScript III kit (Invitrogen). The oligonucleotide primers used were 5′-TCCAGCTGCGCATCTCCTGC-3′ (sense) and 5′-CTTCTC CTTGCACAAAACTC-3′ (antisense) for IL-1*β* (the target was 354 bp in length), and 5′-CGAGACCACCTTCAA CTCGATC-3′ (sense) and 5′-CTTCTGCATGCGGTCGG-3′ (antisense) for *β*-actin (122 bp in length). A total volume of 20 *μ*L of PCR mixture included 10 *μ*l of polymerase chain reaction (PCR) Master Mix (Applied Biosystems, Life Technologies, Carlsbad, CA, USA), 10 pmoles of specific sense and antisense primers for each cytokine gene, and 10 ng of first-strand cDNA. After beginning with a single preincubation step at 95°C for 10 min, PCR was performed under the following conditions: denaturation for 1 min at 94°C, annealing for 1 min at 55°C, and extension for 1 min at 72°C in a thermal cycler (G-STORM, UK). The numbers of cycles used for IL-1*β* and *β*-actin amplification were 38 and 35, respectively. The PCR products were analyzed with 2% agarose gel electrophoresis and ethidium bromide staining and captured using a Kodak molecular imaging system (NY, USA). The relative intensities of the bands were quantified using Gel-Pro image analysis software, version 3.1 (Media Cybernetics, Rockville, Maryland).

### 2.9. Statistical Analysis

All values are expressed as means ± SD, and differences between groups were analyzed by one-way analysis of variance with a Bonferroni multiple comparison test. A *p* value of <0.05 was considered to be statistically significant.

## 3. Results

### 3.1. Expression and Quantification of Recombinant LZ8 by the pNZ8149 Vector and the Host Strain *L. lactis* NZ3900

**Figure 2:**
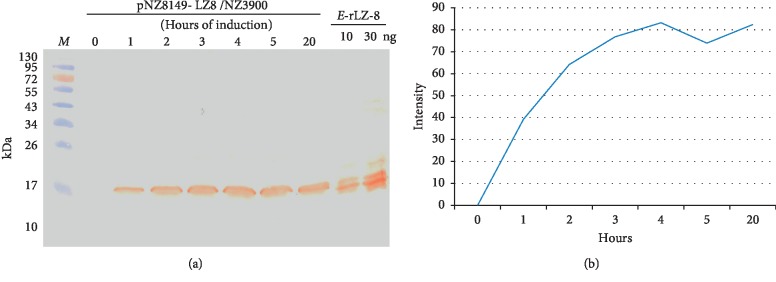
(a) Expression analysis of recombinant LZ8-producing *L. lactis* after nisin induction for 1–20 hours. Immunoblotting of bacterial lysate was performed with lab-made rabbit anti-LZ8-specific antibody. *E. coli*-derived recombinant LZ8 was used as positive control. Lane M, prestained protein markers. (b) Correlation curve of the induction times and the intensity levels of band signals by video densitometer.

cDNA encoding 110 amino acids of LZ8 was inserted into the pNZ8149 vector and expressed in the *L. lactis* strain NZ3900. A band around 15 kDa was confirmed by immunoblotting with lab-made rabbit anti-LZ8 polyclonal antibodies (Figure 2(a)). No signal was detected in the cell extract of wild *L. lactis* NZ3900 strain (data not shown). The optimal yield of nisin-induced rLZ8 in *L. lactis* was 1 *μ*g/2 × 10^10^ colony-forming units for 4 hours according to the intensity levels of standard bands (Figure 2(b)).

### 3.2. Body Weight and Serum Biochemical Data

The average body weight was not significantly different among the three groups of rabbits during the entire experimental period (Table 1). At the beginning of the experiment, there were no significant differences in serum lipid levels among the three groups. However, after the rabbits had been fed with a 2% cholesterol diet for 3 weeks, the levels of total cholesterol and LDL-C in the sera were significantly increased in the three groups and reached an even higher level at the end of 5 weeks. The final levels of total cholesterol and LDL-C at W5 were not statistically different between groups. Moreover, no significant differences were found in HDL-C and triglycerides during the cholesterol feeding period (Table 1). Concerning liver function, serum levels of AST and ALT were significantly higher after 3 weeks of the high cholesterol diet in both the sham and VO groups, but not in the LZ8 group (Table 1). LZ8 appeared to improve liver function by decreasing AST and ALT activities.

### 3.3. Effect of Recombinant LZ8 *L. lactis* on Preventing the Development of Fatty Liver

**Figure 3:**
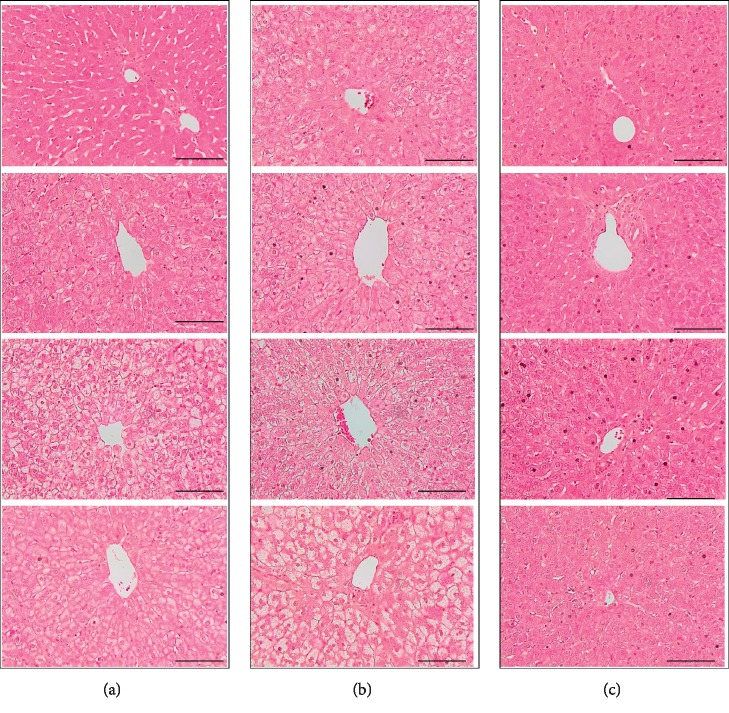
Histological changes in the livers of cholesterol-fed rabbits by H&E staining. Under higher magnification, hepatocyte ballooning and steatosis were observed throughout the sections in the rabbits from the sham and VO groups. H&E staining showed that steatohepatitis was attenuated in rabbits of LZ8 *L. lactis* treatment group. CV, central vein. Scale bar = 100 *μ*m. Magnification ×400. (a) Sham group.(b) VO group. (c) LZ8 group.

In the sham and VO groups, the hepatocellular fat deposition was seen predominantly after 5 weeks of the 2% cholesterol diet (Figure 3, Sham and VO groups). In contrast, lipid droplets were rarely observed in the liver cells from the four rabbits in the LZ group (Figure 3, LZ8 group). Importantly, the results of serum levels of AST and ALT, and H&E staining of livers suggested that the studied medicine, which was composed of recombinant *L. lactis* expressing LZ8, had a liver-protecting effect.

### 3.4. The Effect of Recombinant LZ8 *L. lactis* on Preventing the Development of Early Atherosclerosis

**Figure 4:**
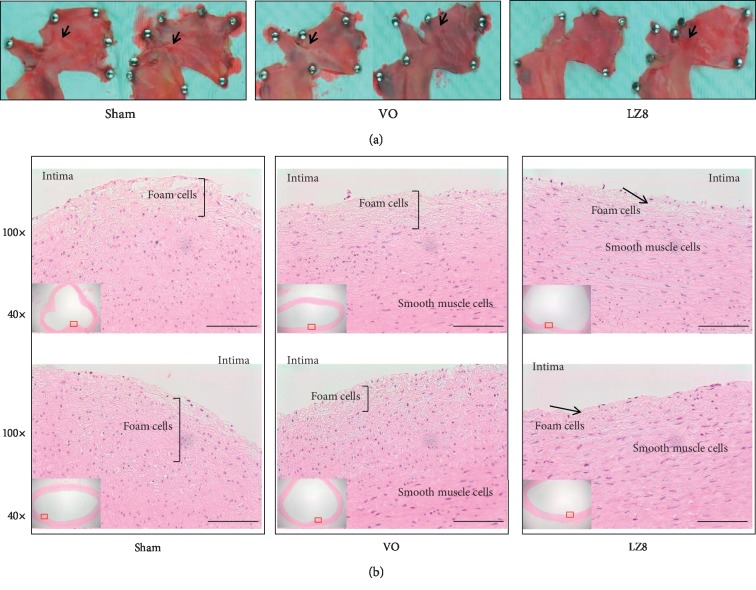
Histological images of aortic arches from three groups of rabbits after 5 weeks of a high cholesterol diet. (a) Sudan IV staining, arrowhead indicates small plaques in fatty streaks were observed, mostly in the aortic arch from sham group. (b) H&E staining, bracket indicates thickness of the intimal layer and arrow indicated foam cell deposition. Scale bar = 100 *μ*m.

To examine the effect of recombinant LZ8 *L. lactis* on the development of atherosclerotic lesions after 5 weeks of the 2% cholesterol diet, the harvested aortic arches were separated into two groups and stained with Sudan red and H&E, respectively. After Sudan IV staining of the six aortas from the three groups of rabbits, atherosclerotic lesions were stained red (Figure 4(a)). As expected, aortic internal surfaces in the sham group showed deposition of lipids, and small plaques in fatty streaks were observed, mostly in the aortic arch (Figure 4(a), panel sham). Compared to the sham group, the plaque areas of the aortic arches in the VO and LZ8 groups were significantly reduced (Figure 4(a), panels VO and LZ8). The other six aortas from the three groups of rabbits were stained with H&E (Figure 4(b)). In the sham and VO groups, the atherosclerotic lesions exhibited a thickened intima. The rabbits in the LZ8 group had a lower intima thickness compared to both the sham and VO groups. These results suggest that oral medicine, which was composed of recombinant *L. lactis* expressing LZ8, had an antiatherosclerotic effect in the rabbit model of atherosclerosis.

### 3.5. Recombinant LZ8 *L. lactis* Reduced the mRNA Expression of IL-1*β* in Cholesterol-Fed Rabbits

**Figure 5:**
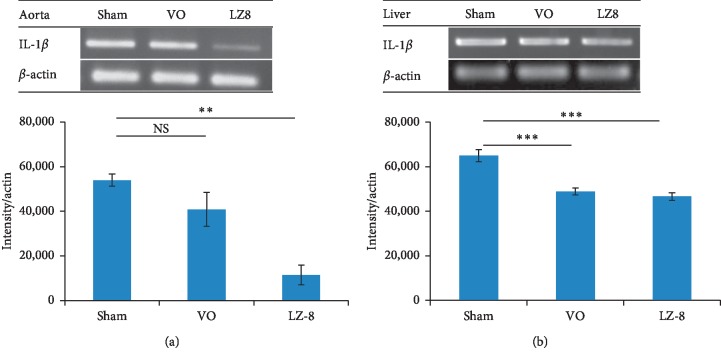
Recombinant LZ8 *L. lactis* vaccine reduced IL-1*β* mRNA expression. (a) Aortas; and (b) livers of high cholesterol-fed rabbits.RT-PCR data were normalized to *β*-actin and presented as mean ± SEM of intensity. ^*∗∗*^*p* < 0.01; ^*∗∗∗*^*p* < 0.001, by one-way analysis of variance with the Bonferroni multiple range test.

Atherosclerosis is an inflammatory disease, and the proinflammatory cytokine IL-1*β* has been well studied as a therapeutic target [18, 19]. The effects of recombinant LZ8 *L. lactis* on the expression levels of IL-1*β* in aortas and liver tissues from the rabbits were evaluated using reverse transcriptase (RT)-PCR. The results showed that the mRNA expression of IL-1*β* was significantly lower in aorta homogenates (Figure 5(a)) and livers (Figure 5(b)) in the LZ group than in the sham group.

## 4. Discussion

Associations between NAFLD and atherosclerosis have been reported in several large epidemiology studies [3, 20, 21]. However, few studies have investigated whether the two diseases share any common pathophysiological mechanisms, which would suggest that they may respond to the same therapy. In the present study, the consumption of a high cholesterol diet for 35 days in a rabbit model resulted in the development of NAFLD and early atherosclerosis. We developed an oral medicine composed of *L. lactis* expressing the LZ8 protein in a nisin-controlled gene expression system and investigated its anti-inflammatory properties. The mucosal administration of therapeutic molecules offers several advantages, such as easy administration and a reduction in adverse effects. Our results showed that the recombinant LZ8 *L. lactis* had a promising anti-inflammatory effect on the development of fatty liver and atheroma in cholesterol-fed rabbits.

Our findings further demonstrated that under a continuous high cholesterol diet, serum total cholesterol, LDL-C, and triglycerides remained at high levels. This pattern of dyslipidemia is commonly seen in real-world atherosclerotic patients with a poor diet or receiving medication but with a poor response to lipid-lowering treatment. The LDL-C deposited in the subendothelial space became oxidized LDL, resulting in severe inflammatory processes. IL-1*β* is an important proinflammatory cytokine that has been associated with the pathogenesis of vascular and immune diseases [22, 23]. In the current study, the aorta expression of IL-1*β* was significantly suppressed after LZ8 treatment. Moreover, H&E staining of the aorta showed that the intima-medial thickness was decreased and the number of foam cells was significantly reduced in the subendothelial space. In the Canakinumab Anti-inflammatory Thrombosis Outcomes Study (CANTOS) trial [24], the use of a highly selective IL-1*β* monoclonal antibody (canakinumab) to inhibit inflammation in patients with atherosclerosis led to a significantly lower rate of recurrent cardiovascular events, without inducing any change in the lipid profile. This result suggests that anti-inflammatory therapy may play an important role in treating atherosclerosis, although the molecular mechanism remains unclear. Methotrexate is a nonselective IL-1*β* inhibitor, and it has been shown to suppress inflammation by diminishing cytokine production [25]. In the Cardiovascular Inflammation Reduction Trial [26], low-dose methotrexate was used for secondary prevention in patients with previous myocardial infarction or multivessel coronary disease. The results showed that methotrexate did not reduce levels of IL-1*β*, IL-6, or C-reactive protein and did not improve cardiovascular events. These clinical drug trials suggest that only specific inhibition of IL-1*β* effectively reduces inflammation and progression of atherosclerosis.

The beneficial functions of LZ8, which is also a bioactive component of *Ganoderma lucidum*, have been reported in several *in vitro* and *in vivo* studies. For example, Hsu et al. showed that LZ8 could suppress intestinal inflammation via a CD45-dependent signaling pathway in a mouse model [27]. In addition, You et al. reported that LZ8 may bind to a specific type of N-glycan that suppresses tumor growth and migration of HCC413 cells [26], and Liang et al. reported that *Pichia*-expressing recombinant LZ8 could induce autophagic cell death in human gastric carcinoma [28]. Furthermore, the oral administration of recombinant LZ8 has been shown to accelerate wound healing in rat livers after monopolar electrosurgery [13]. Recently, Chen et al. demonstrated that LZ8 could reduce nitric oxide synthase and toll-like receptor-4 and suppress the expression of NF-*κ*B in a murine cell line [29].

Recent reports have shown that inflammation plays an important role in the initiation of NAFLD and atherosclerosis. In the present study, we further demonstrated that LZ8 inhibited the expression of IL-1*β* in the liver, decreased fat droplet deposition and infiltration of inflammatory cells, and improved liver function by decreasing liver enzymes (AST, ALT). However, there were several limitations to this study. First, although feeding rabbits with a high cholesterol diet is a well-established animal model for atherosclerotic heart disease, only a few studies have used it as a model for NAFLD [30]. The rabbits' levels of serum cholesterol and triglycerides increased rapidly after consuming cholesterol. However, atherosclerotic patients seldom have a serum cholesterol level over 1000 mg/dL or an LDL level above 500 mg/dL. Thus, rabbits fed with a 2% cholesterol diet may exhibit physiological phenomena that are somewhat different from those observed in real-world atherosclerosis in human subjects. Second, this preliminary study is the first to demonstrate that the administration of LZ8 protein in an animal model could inhibit NAFLD and early atherogenesis within 5 weeks. Further studies are required to investigate the long-term benefits or adverse effects of this treatment. In future studies, we will include 6–10 animals in each group of rabbits in order to obtain more definitive results.

## 5. Conclusions

Taken together, our findings suggest that the anti-inflammatory effect of Lactococcus-expressing LZ8, which is available as a safe and low-cost food, might be a promising medicine for improving both atherosclerosis and NAFLD.

## Figures and Tables

**Table 1:** Effects of oral recombinant *Lactococcus lactis* in high cholesterol diet of rabbits on body weight and serum parameters.

Measurement	Sham			VO			LZ8		
	W0	W3	W5	W0	W3	W5	W0	W3	W5
Body weight (Kg)	2.62 ± 0.32	2.75 ± 0.25	2.79 ± 0.26	2.38 ± 0.30	2.51 ± 0.23	2.61 ± 0.38	2.58 ± 0.25	2.72 ± 0.29	2.83 ± 0.23
Cholesterol (mg/dL)	38 ± 8.6	1264 ± 214^*∗∗*^	1487 ± 552^*∗∗∗*^	41.5 ± 22.9	831.3 ± 511.3^*∗*^	1342 ± 258^*∗∗∗*^	39.3 ± 8.1	1390 ± 602^*∗∗*^	1980 ± 394.7^*∗∗∗*^
HDL-chol (mg/dL)	19 ± 5.8	30 ± 7.9	31.5 ± 11.2	14.2 ± 4.9	39.2 ± 11.9	26.5 ± 11.7	20.5 ± 4.7	29.25 ± 8.7	21.8 ± 5.6
LDL-chol (mg/dL)	8.5 ± 3.6	527.9 ± 80.9^*∗∗*^	540.8 ± 237.5^*∗∗*^	9.85 ± 7.9	291.3 ± 198.8^*∗*^	520.5 ± 96.1^*∗∗∗*^	7.05 ± 2.2	558.1 ± 262.3^*∗∗*^	773.2 ± 121.9^*∗∗∗*^
Triglyceride (mg/dL)	56.5 ± 14.5	89.5 ± 68.3	111.5 ± 52.8	44 ± 14.6	79.8 ± 58.5	71.3 ± 28.7	45.8 ± 5.3	79 ± 44.0	110.8 ± 64.9
*AST (U/L)*	24.2 ± 9.1	348.5 ± 156.9^*∗∗*^	107.5 ± 57.1	18 ± 5.6	179.5 ± 40.9^*∗∗∗*^	52 ± 25.5	36 ± 18.9	83.3 ± 73.8	73.2 ± 29.2
*ALT (U/L)*	44.3 ± 22.6	304 ± 94.2^*∗∗∗*^	134 ± 59.7	33.5 ± 5.8	183.7 ± 58.7^*∗∗∗*^	78.5 ± 25.8	54 ± 22.8	84.3 ± 47.9	98.3 ± 57.5

HDL, high-density lipoproteins; LDL, low-density lipoproteins; AST, aspartate aminotransferase; ALT, alanine aminotransferase.Values are means ± SD of 4 rabbits from each group. The statistics show comparisons between W0 and W3 or W0 and W5 from each group by the Bonferroni multiple range test. ^*∗*^*p* < 0.05; ^*∗∗*^*p* < 0.01; ^*∗∗∗*^*p* < 0.001.

## Data Availability

All data used to support the findings of this study are included in the article.

## Supplementary Materials

Supplementary Materials Supplement Figure 1: alignment of nucleotide sequences of modified Ganoderma lucidum immunomodulatory protein (DMR415LZ8) and GenBank no. M58032.1 by MUSCLE version 3.8. Sequences are aligned with translated region and gaps (dashes) are introduced to optimize alignment. ^*∗*^represents homology. The similarity between them is 70.57%.
