# Supplementary figures and images for: Recombinant Lactococcus lactis Expressing Ling Zhi 8 Protein Ameliorates Nonalcoholic Fatty Liver and Early Atherogenesis in Cholesterol-Fed Rabbits
Source: Biomed Res Int. 2020 Jan 26;2020:3495682. doi: 10.1155/2020/3495682 (PMC7007749; doi:10.1155/2020/3495682)

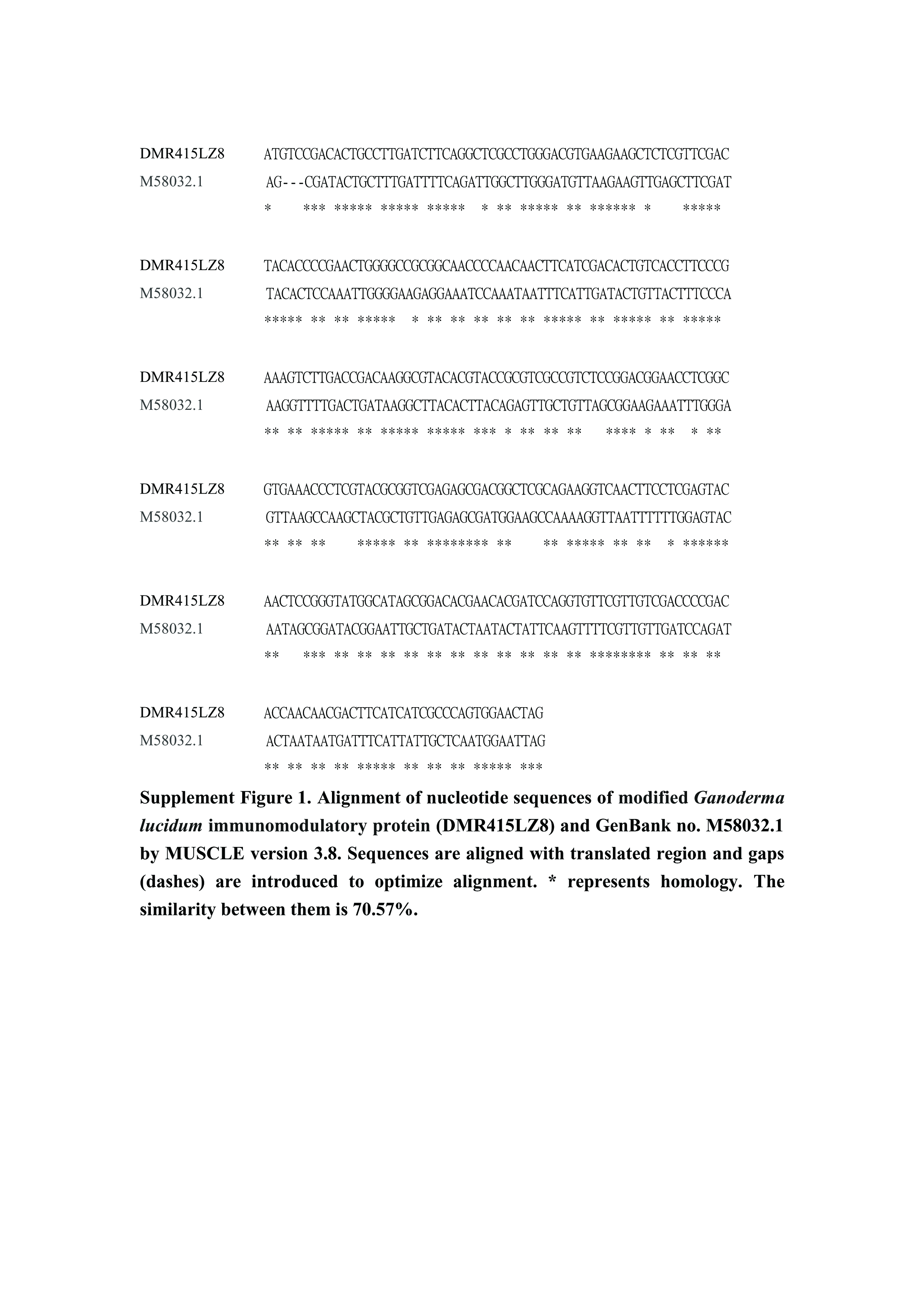

Supplement: Supplementary Materials — Supplement Figure 1: alignment of nucleotide sequences of modified Ganoderma lucidum immunomodulatory protein (DMR415LZ8) and GenBank no. M58032.1 by MUSCLE version 3.8. Sequences are aligned with translated region and gaps (dashes) are introduced to optimize alignment. ∗represents homology. The similarity between them is 70.57%. [file 3495682.f1.tif]
